# Cervical Cancer Screening Awareness and Uptake among Female Health Sciences Students at Islamic University in Uganda

**DOI:** 10.24248/eahrj.v9i2.847

**Published:** 2025-12-24

**Authors:** Mahmud Sulaiman Ishaq, Annet Amadrio, Abdille Said Mohamed, Najjemba Aminah Sembatya, Farah Amal Fandhe, Aremu Babatunde Abdulmujeeb

**Affiliations:** a Islamic University in Uganda

## Abstract

**Background:**

Cervical cancer remains a leading cause of mortality among women in Uganda. This study assessed awareness and uptake of cervical cancer screening among female health science students at the Islamic University in Uganda.

**Methods:**

A cross-sectional study was conducted among 189 female students using a pretested, self-administered questionnaire. Data were analyzed using SPSS version 21.

**Results:**

While 84.7% of respondents were aware of cervical cancer, only 57.7% had acceptable knowledge of symptoms, and merely 11% had ever been screened.

**Conclusion:**

Despite high awareness, cervical cancer screening uptake is very low. Targeted educational and screening interventions are urgently needed.

## BACKGROUND

Cervical cancer is a sexually transmitted viral infection mainly caused by the Human Papilloma Virus (HPV), which attacks the female reproductive tract, causing lacerations and abnormal cervical cells.^1^ Globally, it is estimated that cervical cancer is the 4^th^ most common cancer among women worldwide, with an estimated 604,000 new cases and 342, 000 deaths in 2020, with 90% of the deaths taking place in developing regions like Africa and Southern Asia.^2^

Other risk factors for cervical cancer include early age of first intercourse, multiple sexual partners, HIV infection, smoking, using birth control pills and higher parity, among others.^3^ Cervical cancer is easily preventable, yet a lack of awareness (knowledge) about the disease and its risk factors affects the decision to be screened and vaccinated for cervical cancer.^4^

According to the American Cancer Society (2020), cervical cancer is a leading cause of death among American women, with about 36,000 dying from advanced stages of the disease annually.^5^ About 90% of cervical cancer can be prevented if all women are aware and comply with high-quality cytological screening programs.^6^ Papanicolaou Cytological Testing (Pap smear) allows cervical lesions to be detected before they become cancerous, thereby effectively reducing the incidence of cervical cancer by 75 to 90%.^7^

In Africa, where resources for prevention, diagnosis and treatment are limited or non-existent, cervical cancer imposes a heavy toll on the health, economic and social burdens to women of reproductive age.^8^ The World Health Organization estimates that HPV infections cause approximately 68,000 cases of cervical cancer each year in Africa.^2^ Studies have found that there is a strong interplay between the knowledge of the disease and cervical cancer prevention, which impacts its adoption even when the facility is available.^9^

In East Africa, countries such as Tanzania and Kenya continue to report high incidence and mortality rates from cervical cancer, largely due to late presentation and limited awareness among young women aged 18 to 25 years.^9,10^ Many in this age group lack adequate knowledge about warning signs, risk factors, available prevention methods, and the benefits of early detection, including HPV vaccination and screening.^11^

Uganda has set a strategic goal to reduce 15% of cervical cancer-related deaths by 2025. This ambitious plan aims to reach 50% of the population with prevention awareness information on vaccination against HPV, reduction of exposure to HPV infections and ensure 80% coverage of Visual Inspection with Acetic Acid (VIA) to detect precancerous cervical lesions among women aged 15 to 25 years.^12^ Despite these high statistics, only 33% of women are screened for the disease due to a lack of awareness about the disease symptoms, its risk factors, facilities where to screen and procedures involved in screening, such as papilionaceous smear test and visual Inspection with Acetic Acid, among others.^13^

Female students in health sciences are expected to have better access to medical information and are future health professionals who will influence community health behaviours. However, several studies show that even among health-related students, awareness, attitudes, and uptake of cervical cancer screening remain suboptimal.^14^ Assessing their knowledge and screening practices is therefore essential, as this group plays a critical role in promoting preventive health measures in Uganda. Understanding the gaps among these students provides a strategic starting point for designing targeted educational interventions within training institutions.

## METHODOLOGY

### Study Design and Setting

The study was conducted among female students of the Faculty of Health Sciences, Islamic University in Uganda, Kampala Campus. The faculty of Health Sciences at IUIU Kampala Campus specifically has an enrollment of 357 female students undertaking undergraduate courses.

### Study Site & Study Population

Given that the rate of cervical cancer screening uptake among female students in the Faculty of Health Sciences-IUIU, Kampala Campus was not known, the minimum sample size was calculated using the Yamane formula, ^15^ to determine the sample size from a given population. According to Islamic University in Uganda, the female students in the Faculty of Health Sciences were 357.

### Sample Size

n= N / (1+N (e) ^2^)

Where:

n= signified the sample size
N= signified the population under study (357)
e= signified the margin error (0.05)

Therefore:

n= 357/(1+357 (0.05)^2^)
n= 357/(1+357 (0.0025)
n= 357/(1+0.8925)
n= 357/1.8925
n= 188.64 ≈ 189 respondents

Therefore, the sample size was 189 female students.

From this, a sample size of 189 was obtained using the Taro Yamane formula. The participants participated in the study by completing a well-structured online questionnaire consisting of 3 sections: Demographic characteristics of the respondents, Awareness about cervical cancer and its screening, and Uptake of cervical cancer screening.

### Sampling Technique and Data Collection

The study was conducted for a period of 3 months, from September to November 2022.

Participants were selected using a systematic sampling technique. The student register served as the sampling frame, and a sampling interval of 2 was applied. This meant that after listing all eligible female students, every third female student on the register was selected to participate. If the selected student declined participation or was unavailable, the next immediate eligible female student (the fourth name) was approached as a replacement. It was strictly carried out with only female students who agreed to participate and were recruited by giving written informed consent. Participants were informed about the study's objectives, and their duly filled informed consent form was obtained, ensuring confidentiality. The collected data were entered into a database created using SPSS Inc. 20 statistical software. Data was entered into a Microsoft Excel 2013 spreadsheet for analysis and then presented in tables, figures and statistical texts based on the study objectives. An introductory letter was obtained from the Dean Faculty of Health Sciences at the Islamic University in Uganda (IUIU). More so, approval from the research committee of Islamic University in Uganda, was sought.

To get participants to respond, the survey questions were kept precise and simple, and the questionnaire was electronic and easy to access on any electronic device. A friendly reminder was also sent to class groups, always by the class coordinator.

## RESULTS

### Socio-demographic Characteristics of Participants.

Most participants were in their final year (32.3%). A large majority were single (85.2%), with only 12.7% married. About 69% were aged 21 to 25. While the group included several nationalities, Nigerians formed the largest share at 33.9%, and a small minority, 2.1%, came from Egypt, Comoros, or Cameroon as shown in Table 1.

**TABLE 1:** Socio-Demographic Characteristics of Participants

	Frequency	Percentage (%)
Age (in years)		
18–20	30	15.9
21–25	131	69.3
26–30	26	13.8
31–35	2	1.1
Marital Status		
Married	24	12.7
Single	161	85.2
Others	4	2.1
Nationality		
Nigerian	64	33.9
Kenyan	35	18.5
Somali	32	16.9
Ugandan	54	28.6
Others	4	2.1
Year of Study		
Final year (Year 5 medicine & year 4 nursing)	61	32.3
Year 4	26	13.8
Year 3	31	16.4
Year 2	54	28.6
Year 1	17	8.9

### Awareness about cervical cancer and its screening among female university students

The study showed that 84.7% of respondents had heard of cervical cancer, with lectures and ward teachings serving as the main information source (65.6%), while only 4.8% learned through social media. Most participants (78.8%) correctly identified a virus as the cause, whereas only 1.6% attributed it to parasites.

Regarding symptoms, 57.7% recognised abnormal vaginal bleeding, post-coital bleeding, and foul-smelling discharge, while 18.5% mentioned only foul-smelling discharge and 9% reported no knowledge of symptoms. In terms of prevention, 74.6% cited HPV vaccination and routine screening, 29.6% mentioned smoking cessation, and 4.8% were unsure.

For screening initiation, 42.8% correctly indicated age 21 years, whereas 9% believed screening should start at 40 years, as shown in Table 2.

**TABLE 2:** Shows Whether Respondents Had Ever Heard About Cervical Cancer and Their Source of Information on Cervical Cancer Screening

Variables	Responses	Frequency	Percentage (%)
Awareness about symptoms of cervical cancer	Abnormal vaginal bleeding	54	28.6
	Postcoital bleeding	39	20.6
	Foul-smelling vaginal discharge	35	18.5
	All of the three symptoms	109	57.7
	Don't know	17	9
awareness about how a person can be prevented from getting cervical cancer	Avoid multiple sexual partners	106	56.1
	Avoid early sexual intercourse	84	44.4
	HPV vaccination and routine screening	141	74.6
	Quit cigarette smoking	56	29.6
	Routine screening	97	51.3
	Don't know	9	4.8
awareness on the age of cervical cancer screening should be started	Below 20 years	57	30.2
	At 30 years	34	18
	At 40 years	17	9
	At 21 years	81	42.8

### Uptake of cervical cancer screening among female University students

The study showed that 89% (168) of respondents had never been screened for cervical cancer, while only 11% (21) had ever undergone screening. Among those screened, nearly half (47.6%) were screened at a Regional or National Referral Hospital, whereas 19% were screened at a Health Centre. The most commonly used screening method was Visual Inspection with Acetic Acid (VIA), reported by 71.4% of screened respondents. A smaller proportion underwent Pap smear testing (23.8%), and only 4.8% were screened using Visual Inspection with Lugol's Iodine (VILI), as shown in Table 3.

**TABLE 3:** Uptake of Cervical Cancer Screening Among Female University Students

Variables	Responses	Frequency	Percentage (%)
Ever been screened for cervical cancer	Yes	21	11
	No	168	89
Place of screening			
	Regional/National Referral Hospital	10	5.2
	General/District Hospital	7	3.7
	Health center	4	2.1
	Not Screened	168	89
Screening Methods	Visual inspection with Acetic Acid	15	71.4
	Visual inspection with Lugol's Iodine	1	4.8
	Pap smear test	5	23.8
	HPV testing	0	
	Not Screened	168	

## DISCUSSION

### Awareness levels

The study found that more than half of the participants, 131 (69.3%) were aged 21 to 25 years, indicating that most respondents were young adults within the sexually active age bracket. This age distribution is comparable to the Ethiopian findings by Getaneh et al.^15^, where the majority 217 (54%) of the 403 respondents were also aged 21 to 25 years. However, it differs from the Nigerian study by Omorogbe and Ehizemwogie^16^, which reported that most respondents (46.7%) were between 18–20 years. This variation may be attributed to differences in class composition, as our sample included more senior (clinical-year) students. In terms of marital status, the current study found that 161 (85.2%) of respondents were single, consistent with the Nigerian study (85.3%) ^16^ and the Ethiopian study (96.8%), ^15^ demonstrating a similar demographic trend among university female students in African settings.

Regarding awareness of cervical cancer, the present study established that 160 (84.7%) of students had ever heard of cervical cancer. This high awareness aligns with Jaglarz et al,^4^ who similarly found satisfactory awareness among female university students, although their study noted gaps in understanding HPV as the causative agent. In our study, 149 (78.8%) correctly identified HPV as the cause of cervical cancer, showing stronger aetiological knowledge compared to Dhodapkar et al,^17^ in South India, where only 18.7% of students knew that HPV causes cervical cancer. This difference may be explained by the fact that most of our respondents were in clinical years, giving them greater exposure to reproductive health content.

### Knowledge gaps

Knowledge of symptoms was moderate, with 109 (57.7%) identifying abnormal vaginal bleeding, post-coital bleeding, and foul-smelling discharge as symptoms. This is consistent with the Ethiopian findings where 52% demonstrated similar symptom knowledge.^15^ Conversely, it contrasts with the study in Thailand,^18^ where only 34% recognized common symptoms of cervical cancer. Again, this difference may be attributed to variations in curriculum emphasis and exposure to clinical practice.

Awareness of prevention was relatively satisfactory, with 141 (74.6%) identifying HPV vaccination and routine screening as key preventive strategies. This contradicts findings from Abdullahi et al, ^19^ in Qatar, where 75.6% of female students lacked knowledge about screening and vaccination. The discrepancy may be due to differences in health-science training, as the majority of our participants were in advanced clinical years.

### Comparison with other studies

Despite the relatively strong awareness indicators, uptake of cervical cancer screening remained very low in this study (11%), mirroring the low uptake reported by Amosu et al,^20^ in Nigeria (89.1% had never been screened). This persistent pattern across contexts suggests that awareness alone does not translate into screening behaviour. Among the few students who were screened, nearly half (47.6%) underwent screening in regional or national referral hospitals, consistent with findings from a study done in Malawi,^21^ where hospital-based recommendations influenced screening uptake. Additionally, 71.4% of screened students in the present study underwent VIA, consistent with typical screening practices in low-resource settings, although the pattern differed from findings by Twinomujuni et al,.^22^ in Uganda, who reported low Pap smear utilization among female students.

### Limitations of the Study

The study was conducted among uniquely selected students from IUIU-Kampala Campus, which had unique characteristics from other sister campuses, thus its findings could not be fully representative of students on all the campuses. Some students were not willing to participate due to their busy academic schedules and the belief that the study would waste their academic time. The data collected in the study was based on self-reported responses, which may have introduced biases or inaccuracies in the results. Respondents may have provided socially desirable answers or may not have accurately recalled their knowledge. The study used a cross-sectional design, which means that data was only collected at one point in time. A longitudinal design, where data is collected over some time, would provide more insights into changes in Awareness and uptake of screening over time. Nevertheless, the study adds to the existing literature on knowledge and uptake of cervical cancer screening among university students, particularly in sub-Saharan Africa. The findings of the study may be useful in developing targeted interventions to improve awareness and uptake of cervical cancer screening among university students.

## CONCLUSION

This study demonstrated that although awareness of cervical cancer was relatively high among female health sciences students, the actual uptake of cervical cancer screening remained remarkably low. Notable knowledge gaps persisted regarding symptoms, prevention, and treatment, indicating that awareness does not automatically translate into informed decision-making or screening behavior. These findings underscore the need for more targeted and context-specific educational efforts within university settings.

The broader implications highlight the importance of strengthening cancer-prevention competencies among future health professionals, as their knowledge and attitudes directly influence community health promotion. Improving understanding of cervical cancer screening among students contributes to national efforts aimed at increasing early detection and reducing cervical cancer morbidity and mortality.

Practically, universities should integrate cervical cancer education into routine student health programs, establish on-campus screening outreach through partnerships with local health facilities, and ensure easy access to accurate information on available screening methods. Tailored awareness campaigns, peer-led education, and involvement of clinical instructors could further enhance uptake.

Future research should explore barriers to screening among health sciences students, assess the effectiveness of university-based interventions, and compare trends across different faculties and campuses. Longitudinal studies may also provide deeper insight into how knowledge and screening practices evolve over the course of students’ training.
